# Dietary Chlorogenic Acid Attenuates Liver Injury, Lipid Metabolic Disturbance, and Inflammatory Responses in Broilers Under High Stocking Density

**DOI:** 10.3390/ani16162552

**Published:** 2026-08-15

**Authors:** Dongying Bai, Yi Zhang, Bo Zheng, Fangshen Guo, Wenrui Zhen, Ziyue Zhao, Keyuan Lei, Chenjie Zhang, Cai Zhang, Yushu Zhang, Bingkun Zhang, Yanbo Ma

**Affiliations:** 1 Department of Animal Physiology, College of Animal Science and Technology, Henan University of Science and Technology, Luoyang 471003, China; 9900427@haust.edu.cn (D.B.); zhangyi439250@gmail.com (Y.Z.); zhengbo@stu.haust.edu.cn (B.Z.); gfshen@haust.edu.cn (F.G.); zhenwenr@126.com (W.Z.); 240320181095@stu.haust.edu.cn (Z.Z.); leikeyuan@stu.haust.edu.cn (K.L.); 240320181063@stu.haust.edu.cn (C.Z.); zhangcai@haust.edu.cn (C.Z.); 2 Henan International Joint Laboratory of Animal Welfare and Health Breeding, College of Animal Science and Technology, Henan University of Science and Technology, Luoyang 471000, China; 3 Innovative Research Team of Livestock Intelligent Breeding and Equipment, Science & Technology Innovation Center for Completed Set Equipment, Longmen Laboratory, Luoyang 471023, China; 4 Department of Agriculture, Graduate School of Science and Technology, Shinshu University, Matsumoto 399-4511, Japan; 23hs552b@shinshu-u.ac.jp; 5 State Key Laboratory of Animal Nutrition, Department of Animal Nutrition and Feed Science, College of Animal Science and Technology, China Agricultural University, Beijing 100193, China; bingkunzhang@126.com

**Keywords:** NADPH oxidase 5, AMP-activated protein kinase, arachidonic acid metabolism, oxidative damage, phytogenic feed additive

## Abstract

This study evaluated whether chlorogenic acid (CGA) attenuates hepatic injury in broilers under high stocking density (HSD). A total of 216 experimental male Arbor Acres broilers were assigned to normal density without CGA (ND; 14 birds/m^2^), high density without CGA (HD; 22 birds/m^2^), normal density with 0.1% CGA (NDCGA), or high density with 0.1% CGA (HDCGA) groups, with six cages per treatment. CGA was supplied from day 7 to 42. At 42 d, live-weight densities were 35.00, 50.25, 34.48, and 49.38 kg/m^2^, respectively. Compared with their corresponding normal-density groups, the high-density groups had lower body weights at 21, 28, 35, and 42 d, and lower average daily gain (ADG) during days 21–42 (*p* < 0.05), whereas CGA did not significantly alter body weight or ADG within either stocking density (*p* > 0.05). At 35 and 42 d, the HD group had lower hepatic T-AOC, SOD, and CAT activities and higher MDA content than the ND group (*p* < 0.05). Compared with the HD group, the HDCGA group had higher T-AOC and CAT activities and lower MDA content at both ages, and higher SOD activity at 42 d (*p* < 0.05). Qualitative histological and transcript-level alterations were evident at 28 d. Compared with HD, the HDCGA group showed treatment-associated shifts in AMPK, SREBP1, ACACA, PLA2G4A, COX-2, ALOX5, and NOX5 mRNA abundance (*p* < 0.05). Under the experimental conditions, 0.1% CGA was associated with partial attenuation of HSD-related hepatic oxidative, lipid-metabolic, and inflammatory disturbances without a significant growth response.

## Simple Summary

High stocking density can impair broiler health as live-weight load per unit area increases during growth. This study examined whether 0.1% dietary chlorogenic acid (CGA), supplied from 7 to 42 d of age, could support liver health in broilers housed at 14 or 22 birds/m^2^. At 42 d, the recorded live-weight densities were 35.00 and 34.48 kg/m^2^ in the normal-density groups without and with CGA, respectively, and 50.25 and 49.38 kg/m^2^ in the corresponding high-density groups. Compared with their corresponding normal-density groups, the high-density groups had lower body weights and later-stage average daily gains, whereas CGA did not significantly restore growth within either stocking density. Compared with the HD group, the HDCGA group was associated with improved hepatic antioxidant indices and less severe lipid deposition and inflammatory changes in representative liver sections. The molecular findings showed treatment-associated differences in NOX5, SREBP1, ACACA, PLA2G4A, COX-2, ALOX5, and AMPK mRNA abundance. Thus, 0.1% CGA may support hepatic health under high-density conditions, although growth recovery was not demonstrated and only one CGA dose was evaluated.

## 1. Introduction

Modern broiler production increasingly emphasizes high output per unit floor area, and high stocking density (HSD) is therefore frequently used in intensive production systems [1,2,3,4,5]. However, stocking density is most appropriately expressed as total live weight per unit usable floor area (kg/m^2^), because the biological pressure imposed by a fixed number of birds per square meter increases as birds grow. The European Union establishes a general maximum of 33 kg/m^2^, with conditional increases to 39–42 kg/m^2^ when additional environmental, management, and welfare requirements are met [6]. Recent welfare assessments and experimental studies further indicate that increasing live-weight density can restrict movement, impair litter quality, and increase the risks of footpad lesions and other welfare problems [7,8]. Accordingly, the terms normal density (ND) and high density (HD) in the present study describe the lower- and higher-density experimental treatments rather than universal regulatory categories.

The liver is a central metabolic organ in broilers and plays an important role in both the generation and clearance of reactive oxygen species (ROS) [9]. Previous studies have shown that HSD can disrupt redox homeostasis, increase lipid peroxidation, and induce oxidative damage [1,10,11]. Related work has also linked HSD with altered linoleic- and arachidonic-acid metabolism and with increased production of inflammatory lipid mediators, including prostaglandin E_2_ and leukotriene B_4_ [12]. These changes provide a plausible connection among oxidative stress, hepatic lipid accumulation, and inflammatory injury, although the temporal and mechanistic relationships remain incompletely resolved.

Although HSD has been associated with impaired growth, deteriorated environmental conditions, altered behavior, and reduced welfare [2,3,4,5,13,14,15,16], effective nutritional strategies for protecting hepatic metabolic homeostasis under these conditions remain limited. A suitable intervention would ideally reduce oxidative pressure without compromising feed intake or growth and would also limit the downstream lipid-metabolic and inflammatory consequences of chronic density stress.

Chlorogenic acid (CGA) is a hydroxycinnamic acid ester abundant in coffee, tea, fruits, vegetables, and medicinal plants [17]. Its catechol structure supports radical-scavenging and metal-chelating activity [17]. Dietary CGA has shown antioxidant and metabolic benefits in poultry [18,19,20], and experimental studies have linked it to enhanced endogenous antioxidant defense, AMPK-related lipid regulation, and suppression of inflammatory signaling [21,22,23,24,25,26,27,28]. In broilers, CGA has improved antioxidant status and intestinal health under several stress conditions [21,22,24,25,26,27,29]. Nevertheless, its effects on the coordinated hepatic responses to HSD have not been systematically characterized. Therefore, the present study evaluated the effects of 0.1% dietary CGA on growth performance, liver histology, antioxidant capacity, hepatic tissue enzyme activities, transcriptomic profiles, and selected redox-, lipid-, and inflammation-related genes in broilers exposed to HSD.

## 2. Materials and Methods

### 2.1. Animals and Experimental Design

The experiment was conducted at the Animal Research Center of Henan University of Science and Technology. A total of 288 one-day-old Arbor Acres male broilers of similar initial body weight were used. Of these, 216 experimental broilers were randomly assigned to four treatments, with six replicate cages per treatment. The remaining 72 treatment-matched reserve broilers were maintained solely to restore cage population after intermediate destructive sampling. For each treatment, 18 reserve broilers were divided into three age-specific cohorts of six birds, designated for replacement at 21, 28, and 35 d, respectively. Each reserve cohort was maintained in a separate reserve cage with usable floor area adjusted to reproduce the corresponding treatment-specific stocking density (14 birds/m^2^ for ND and NDCGA or 22 birds/m^2^ for HD and HDCGA), while environmental conditions and feeding management were identical to those of the corresponding experimental group. The treatments were normal stocking density with the basal diet (ND), high stocking density with the basal diet (HD), normal stocking density with 0.1% CGA (NDCGA), and high stocking density with 0.1% CGA (HDCGA). Each experimental cage provided 0.50 m^2^ of usable floor area. The ND and NDCGA cages contained 7 birds (14 birds/m^2^), whereas the HD and HDCGA cages contained 11 birds (22 birds/m^2^). Based on the six prespecified experimental-cage records per treatment, the live-weight densities in the ND, HD, NDCGA, and HDCGA groups were 7.82, 11.25, 7.63, and 10.89 kg/m^2^ at 21 d; 15.17, 22.33, 14.77, and 21.61 kg/m^2^ at 28 d; 24.65, 35.65, 24.43, and 35.35 kg/m^2^ at 35 d; and 35.00, 50.25, 34.48, and 49.38 kg/m^2^ at 42 d, respectively. All experimental and reserve birds received the basal diet from day 1 to 6. From 7 to 42 d, the ND and HD experimental groups continued to receive the basal diet, whereas the NDCGA and HDCGA experimental groups received the basal diet supplemented with 0.1% CGA (purity ≥ 99%; Changsha Shanghe Biotechnology Co., Ltd., Changsha, China). The corresponding reserve cohorts received the same treatment-specific diets from 7 d until transfer. Table 1 reports the basal-diet formulation only; the treatment-specific 0.1% CGA supplementation is therefore not included in the basal-diet ingredient totals. Each experimental cage was equipped with an external full-length trough feeder and a nipple-drinker line; the feeding and drinking configuration was identical among cages and did not reduce usable cage-floor area. Thus, 7 birds in the ND/NDCGA cages and 11 birds in the HD/HDCGA cages shared the same standardized cage-level feeding and drinking system. This housing arrangement followed the experimental system used in our previous study [11]. The exact linear feeder space per bird and number of birds per nipple could not be reconstructed reliably from the retained facility records and are therefore not reported retrospectively. Feed and water were provided ad libitum. Room temperature was maintained at 34 °C on day 1, decreased by approximately 1 °C every 2 d until reaching 21 °C, and was then maintained at that level. Relative humidity, health status, and mortality were monitored daily, and routine vaccination and sanitation procedures were followed.

### 2.2. Sample Collection

At 21, 28, 35, and 42 d of age, six broilers were selected from each treatment, with one bird sampled from each replicate cage. Selected birds had a body weight within ±5% of the treatment mean. Sampling was performed between 08:00 and 10:00 without prior fasting. Birds were humanely euthanized by cervical dislocation, the abdomen was opened immediately, and liver tissue was excised within 1 min. Surface blood was removed with pre-cooled sterile saline, and samples were collected from a consistent position in the left hepatic lobe. Tissue for histology was fixed in 4% paraformaldehyde, whereas tissue for RNA sequencing, RT-qPCR, and biochemical analyses was divided into RNase-free tubes, snap-frozen in liquid nitrogen within 30 s, and stored at −80 °C. Following destructive sampling at 21, 28, and 35 d, the six birds in the corresponding treatment- and age-matched reserve cohort were weighed and allocated, one bird to each of the six sampled experimental cages, using the closest body-weight match. This procedure restored the assigned bird number and stocking density in every experimental cage. Each reserve cohort was used only once and was completely transferred at its designated age; therefore, no additional birds were required to replenish the reserve cages. No replacement bird was introduced after the terminal sampling at 42 d. After transfer, replacement birds remained in the corresponding experimental cages and were included in subsequent cage-level body-weight measurements, but they were not selected for later tissue sampling. The age-specific reserve cohorts were used solely as replacement pools and were not treated as experimental replicates.

### 2.3. Growth Performance Measurement

Body weight was recorded at 21, 28, 35, and 42 d for the six prespecified experimental cages per treatment. Only experimental-cage records were included in the statistical analysis; records from the age-specific reserve cohorts were excluded. Average daily gain (ADG) was calculated for days 21–28, 28–35, and 35–42, and the overall verified interval from days 21–42. The replicate cage was the experimental unit. The retained archive did not contain uninterrupted cage-level feed-disappearance records for days 1–42; therefore, overall day 1–42 ADFI and FCR were not reconstructed from phase summaries.

### 2.4. Liver Histology

Liver samples fixed in 4% paraformaldehyde for at least 24 h were dehydrated through graded ethanol, cleared in xylene, embedded in paraffin, and sectioned at 4 μm for hematoxylin and eosin (H&E) staining. For Oil Red O staining, fixed tissue was cryoprotected in 30% sucrose at 4 °C, embedded in optimal cutting temperature compounds, and sectioned at 10–15 μm at −20 °C. Cryosections were rehydrated with phosphate-buffered saline, stained with freshly prepared Oil Red O working solution, differentiated with isopropanol, counterstained with hematoxylin, and mounted with glycerol gelatin. H&E sections were examined at 200× and 400×, and Oil Red O sections were examined at 400×. Images were captured and reviewed using CaseViewer 2.0. The archived histological component was designed for representative qualitative morphological assessment; a prespecified blinded scoring system and a validated quantitative histomorphometric protocol were not included in the original analysis plan. Accordingly, no post hoc quantitative scores were generated from selected representative images.

### 2.5. Hepatic Antioxidant Capacity and Hepatic Tissue Enzyme Activities

For antioxidant analyses, frozen liver tissue was thawed on ice, rinsed with ice-cold physiological saline, blotted dry, and homogenized with nine volumes of pre-cooled 0.9% saline (1:9, *w*/*v*) to prepare a 10% tissue homogenate. The homogenate was centrifuged at 3500 r/min for 10 min at 4 °C, and the supernatant was collected. Total protein was determined using the Coomassie brilliant blue method. Total antioxidant capacity (T-AOC) was measured using an ABTS-based assay, superoxide dismutase (SOD) activity using the WST-1 method, catalase (CAT) activity using the ammonium molybdate method, and malondialdehyde (MDA) content using the thiobarbituric acid method. For hepatic tissue enzyme analyses, a separate 10% liver homogenate was centrifuged at 3000 r/min for 10 min at 4 °C, and aspartate aminotransferase (AST), alanine aminotransferase (ALT), and alkaline phosphatase (ALP) activities were measured in the resulting supernatant using the corresponding colorimetric enzyme assays. All kits were obtained from Nanjing Jiancheng Bioengineering Institute (Nanjing, China), and procedures were performed according to the manufacturer’s instructions. Results were normalized to tissue protein where appropriate.

### 2.6. Hepatic Transcriptome Sequencing and Data Analysis

At 28, 35, and 42 d of age, four independent liver samples from each of the ND, HD, and HDCGA groups were used for RNA sequencing (*n* = 4 biological replicates per group per age; 36 libraries in total), with each sample originating from a different replicate cage. The transcriptomic component focused on the primary ND-versus-HD and HD-versus-HDCGA contrasts; the NDCGA group was not included in RNA sequencing. No biological sample pooling was performed; each library was prepared from an individual liver sample, and equimolar pooling occurred only at the library-sequencing stage. Total RNA was extracted using the RNeasy Mini Kit (Qiagen). RNA concentration, purity, and integrity were assessed using a NanoPhotometer (Implen, Westlake Village, CA, USA), 1% agarose gel electrophoresis, and an Agilent 2100 Bioanalyzer (Agilent Technologies, Santa Clara, CA, USA). Only samples with an RNA integrity number ≥8 were used for library construction. Poly(A) mRNA was enriched from 3 μg of total RNA using oligo(dT) magnetic beads, fragmented to approximately 450 bp, and reverse-transcribed into double-stranded cDNA. Following end repair, A-tailing, adapter ligation, size selection, and 12–15 cycles of PCR amplification, library quality was evaluated with an Agilent 2100 Bioanalyzer and a Qubit fluorometer (Thermo Fisher Scientific, Waltham, MA, USA). Equimolar libraries were sequenced on an Illumina HiSeq 2500 platform to generate 150 bp paired-end reads.

Raw FASTQ reads were processed with Trimmomatic v0.39 to remove adapters, bases with Q < 20, and reads shorter than 36 bp. Read quality was assessed using FastQC v0.10.1. Clean reads were aligned to the chicken reference genome GRCg6a using HISAT2, and gene-level counts were generated with HTSeq-count v0.13.5 in intersection-strict mode. Differential expression was analyzed with DESeq2 v1.24.0. Genes with a false discovery rate (FDR) < 0.05 and a |log_2_ fold change| ≥ 1 were considered differentially expressed. KEGG pathway enrichment analysis was conducted using DAVID. The highest-ranked pathways were visualized using the enrichment *p* value reported by DAVID; pathways with an adjusted *p* value < 0.05 were regarded as statistically significant, whereas other displayed pathways were interpreted descriptively. The retained validated archive did not contain a complete auditable per-library summary of Q20/Q30 values, mapping rates, principal-component-analysis output, or formal batch-assessment records; these metrics were therefore not reconstructed or estimated retrospectively.

### 2.7. Validation by Real-Time Quantitative PCR (RT-qPCR)

RT-qPCR analyses used six independent liver samples per treatment per age (*n* = 6 biological replicates), with one sample originating from each replicate cage. Total RNA was extracted from liver tissue using TRIzol reagent, and RNA purity was evaluated from the A260/A280 ratio. Equal amounts of RNA were reverse-transcribed using the Evo M-MLV Reverse Transcription Kit (Accurate Biology, Changsha, China; AG11728). RT-qPCR was performed on a Bio-Rad CFX Connect system in a 20 μL reaction containing 2 μL of diluted cDNA, 0.4 μL each of forward and reverse primer, 10 μL of 2× SYBR Green Pro Taq HS Premix (Accurate Biology; AG11701), and 7.2 μL of RNase-free water. GAPDH was used as the reference gene, and relative expression was calculated using the 2^−ΔΔCt^ method. PRKAA1 (AMPK), SREBF1 (SREBP1), ACACA, NOX5, PLA2G4A, PTGS2 (COX-2), and ALOX5 were selected because they were differentially expressed or represented central nodes in enriched redox, lipogenic, and arachidonic acid pathways, showed biologically coherent treatment-associated patterns, and permitted reliable gene-specific primer design (Table 2).

### 2.8. Statistical Analysis

Statistical analyses were performed using SPSS 27.0 (IBM Corp., Armonk, NY, USA), with the replicate experimental cage as the experimental unit. Although the treatment structure can be represented as two stocking-density levels and two dietary conditions, the four treatment combinations were prespecified as distinct experimental treatments, and the primary inferential objective was direct comparison between these four combinations. Accordingly, data were analyzed by one-way analysis of variance with treatment as the fixed effect. When the overall treatment effect was significant, Duncan’s multiple-range test was used for pairwise comparisons. Data are expressed as means ± standard error of the mean (SEM), and *p* < 0.05 was considered statistically significant. The age-specific reserve cohorts were used solely to provide treatment-matched replacement birds and were not included as experimental replicates. This analysis evaluates differences among the four treatment combinations and does not provide separate *p* values for stocking-density main effects, CGA main effects, or the stocking density × CGA interaction. Therefore, no factorial main-effect or interaction-dependent conclusion was drawn.

## 3. Results

### 3.1. Growth Performance

Body weight and average daily gain are summarized in Table 3. The HD and HDCGA groups had lower body weights than their corresponding normal-density groups at 21, 28, 35, and 42 d (treatment effect, *p* < 0.05). No significant difference was detected between ND and NDCGA or between HD and HDCGA groups at any age (*p* > 0.05). ADG did not differ among treatments during days 21–28 or 28–35 (*p* > 0.05). During days 35–42 and across the verified day 21–42 interval, high-density groups showed lower ADG than normal-density groups (*p* < 0.05), whereas CGA did not significantly alter ADG within a given stocking density (*p* > 0.05).

### 3.2. Hepatic Antioxidant Indices and Tissue Enzyme Activities

Hepatic antioxidant indices are shown in Table 4. No significant treatment differences were detected at 21 or 28 d (*p* > 0.05). At 35 and 42 d, the HD group had lower T-AOC, SOD, and CAT activity and higher MDA content than the ND group (*p* < 0.05). Compared with the HD group, the HDCGA group showed higher T-AOC and CAT activity and lower MDA content at 35 and 42 d (*p* < 0.05); SOD activity was also higher at 42 d. Within the antioxidant endpoints evaluated, the NDCGA group did not show a consistent adverse treatment-associated pattern relative to ND.

Hepatic tissue enzyme activities are shown in Table 5. At 35 d, the HD group had higher AST, ALT, and ALP activities than the ND group (*p* < 0.05). At 42 d, AST and ALP remained higher in the HD group, whereas the overall treatment effect for ALT approached but did not reach significance (*p* = 0.053). Compared with the HD group, the HDCGA group had lower AST and ALP activities at 35 and 42 d and lower ALT activity at 35 d (*p* < 0.05). Within the hepatic tissue enzyme endpoints evaluated, the NDCGA group did not show a consistent adverse treatment-associated pattern relative to ND.

### 3.3. Histological Changes in the Liver

**Figure 1:**
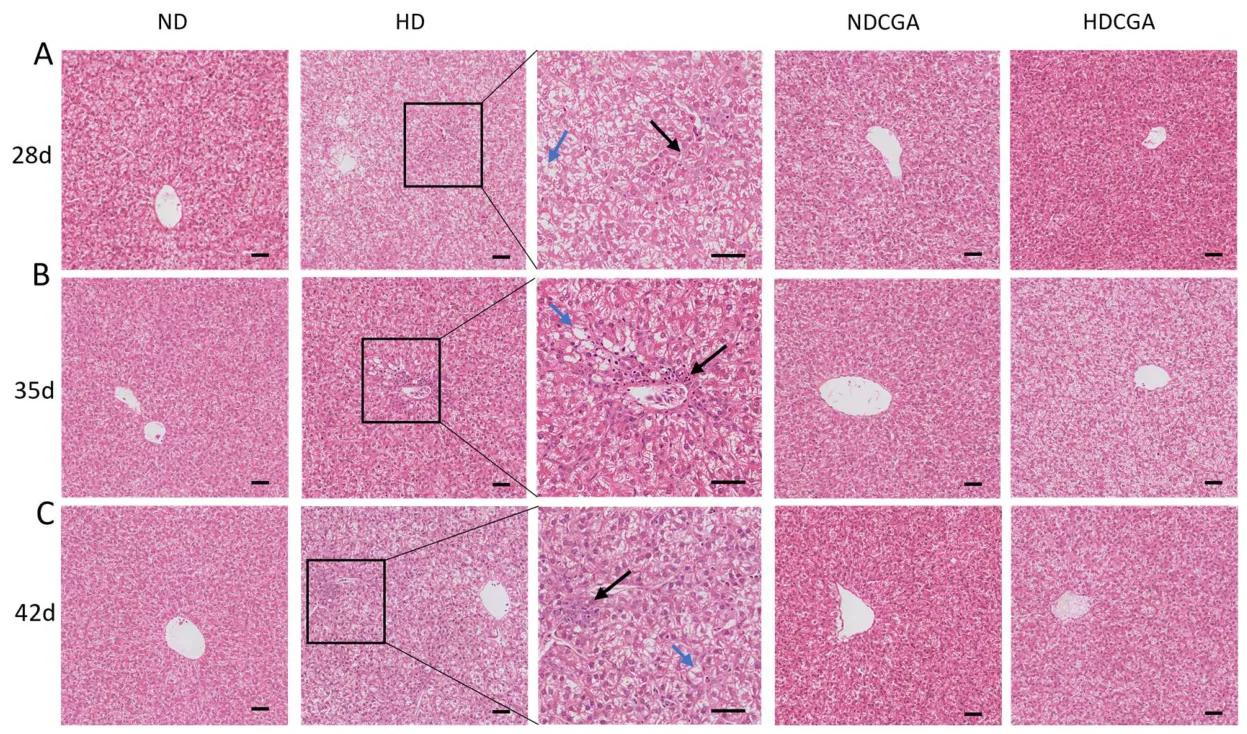
Effects of CGA on hepatic histomorphology in broilers under HSD (H&E staining). Representative sections were obtained at 28, 35, and 42 d (**A**–**C**). Low-magnification images (200×) show overall architecture, whereas high-magnification images (400×) show local details. Blue arrows indicate hepatocellular vacuolation, and black arrows indicate inflammatory cell infiltration. Scale bars: 50 μm (200×) and 20 μm (400×). ND, normal stocking density with the basal diet; HD, high stocking density with the basal diet; NDCGA, normal stocking density with 0.1% chlorogenic acid; HDCGA, high stocking density with 0.1% chlorogenic acid. Representative images are presented for qualitative morphological assessment only; no quantitative histomorphometric inference was made.

**Figure 2:**
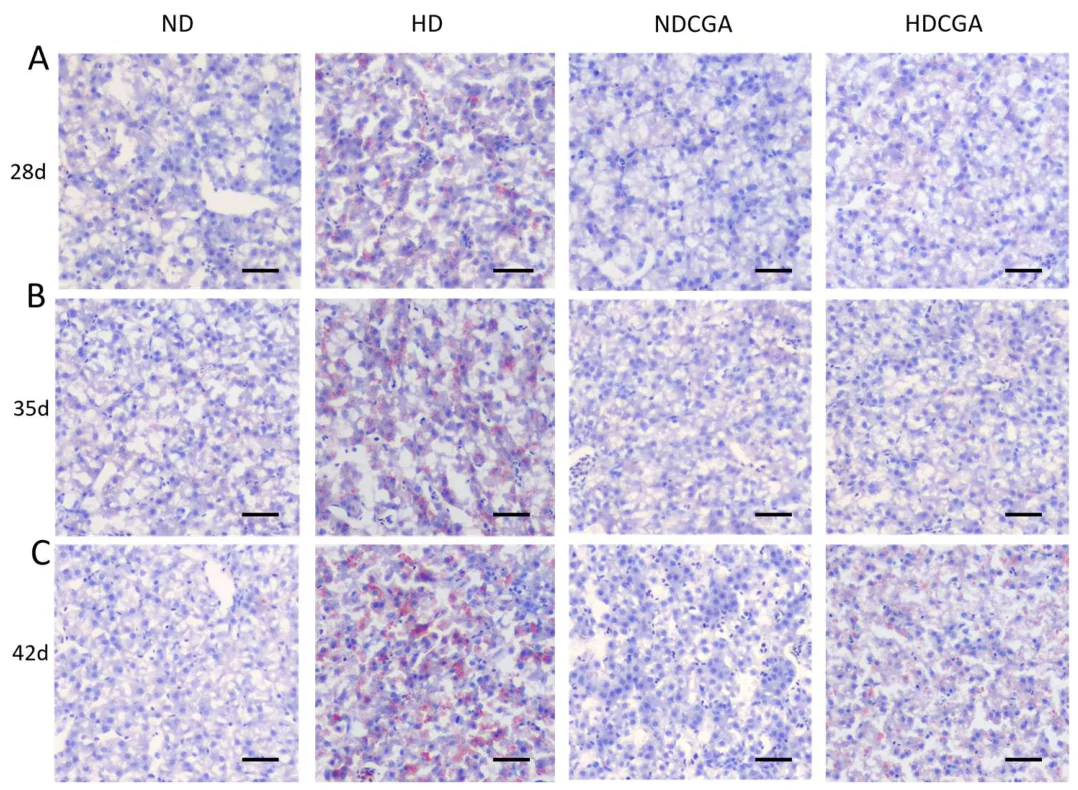
Effects of CGA on hepatic lipid deposition in broilers under HSD (Oil Red O staining). Representative sections were obtained at 28, 35, and 42 d (**A**–**C**). Nuclei are blue and neutral lipid droplets are red. Images were captured at 400×. Scale bar = 20 μm. ND, normal stocking density with the basal diet; HD, high stocking density with the basal diet; NDCGA, normal stocking density with 0.1% chlorogenic acid; HDCGA, high stocking density with 0.1% chlorogenic acid. Representative images are presented for qualitative morphological assessment only; no quantitative histomorphometric inference was made.

H&E and Oil Red O staining were used to evaluate hepatic morphology and lipid deposition (Figure 1 and Figure 2). At 28 d, representative sections from the HD group showed hepatocellular cytoplasmic vacuolation, inflammatory cell infiltration, and visible lipid droplet deposition. These qualitative changes appeared more pronounced at 35 and 42 d. At the corresponding ages, representative HDCGA sections showed better-preserved hepatic architecture, visually fewer lipid droplets, and less inflammatory cell infiltration than HD sections. These observations were qualitative and were not interpreted as quantitative histomorphometric measurements.

### 3.4. Differentially Expressed Gene Analysis

**Figure 3:**
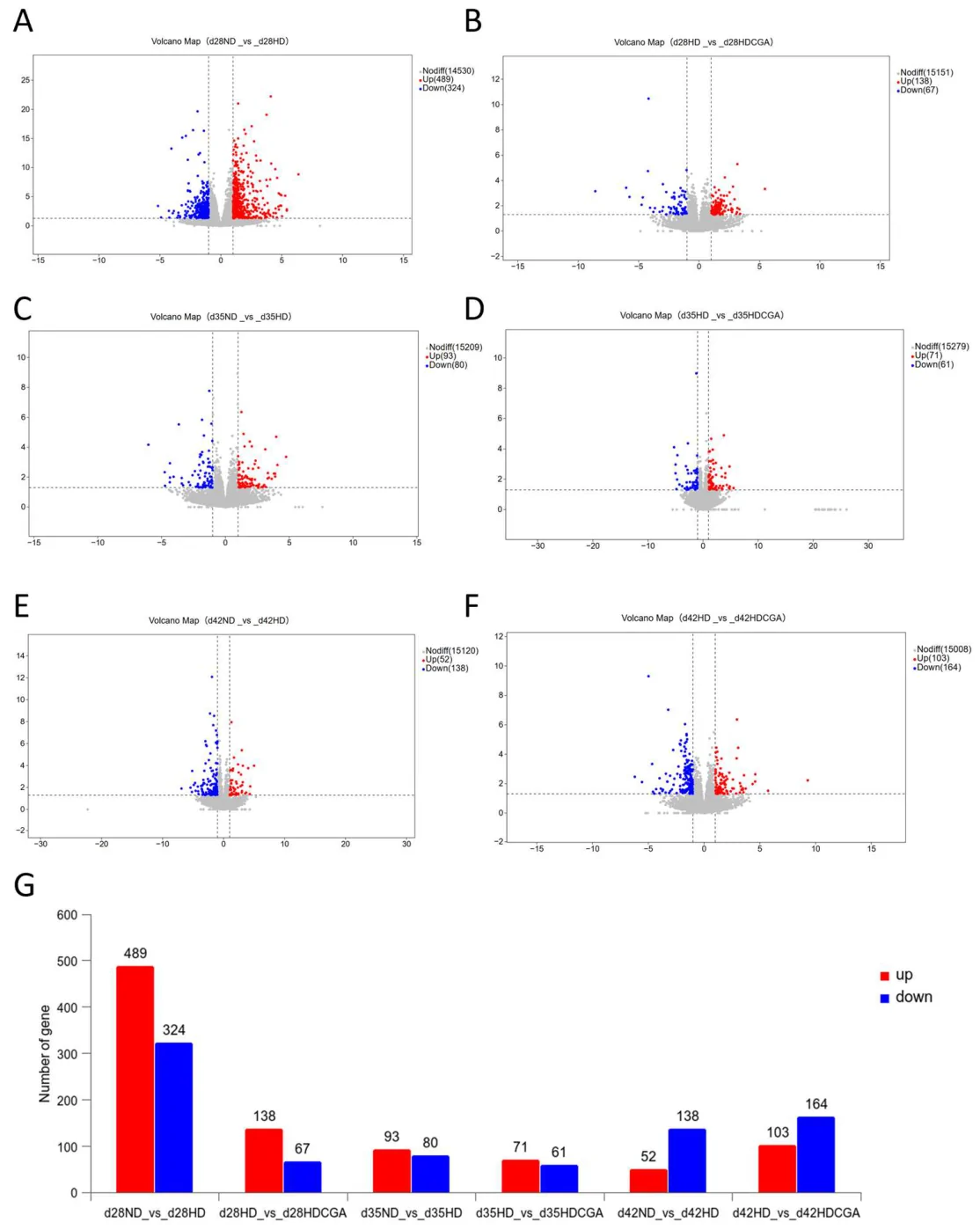
Distribution of hepatic DEGs in broilers under HSD. Panels (**A**–**F**) show volcano plots for ND versus HD and HD versus HDCGA at 28, 35, and 42 d. Red and blue points indicate significantly upregulated and downregulated genes, respectively, based on a FDR < 0.05 and a |log_2_ fold change| ≥ 1; gray points indicate genes that did not meet these criteria. Panel (**G**) summarizes DEG numbers. ND, normal stocking density with the basal diet; HD, high stocking density with the basal diet; HDCGA, high stocking density with 0.1% chlorogenic acid. RNA-seq analyses used *n* = 4 biological replicates per represented group per age.

Transcriptomic sequencing identified marked, age-dependent differences in hepatic gene expression (Figure 3). In the ND versus HD comparison, 813, 173, and 190 differentially expressed genes (DEGs) were detected at 28, 35, and 42 d, respectively. In the HD versus HDCGA comparison, 205, 132, and 267 DEGs were detected at the corresponding ages. Thus, the largest transcriptional response to HSD occurred at 28 d, whereas the greatest number of CGA-responsive genes was observed at 42 d. This temporal pattern indicates that hepatic transcriptional responses to density and CGA changed across growth stages rather than remaining uniform.

**Figure 4:**
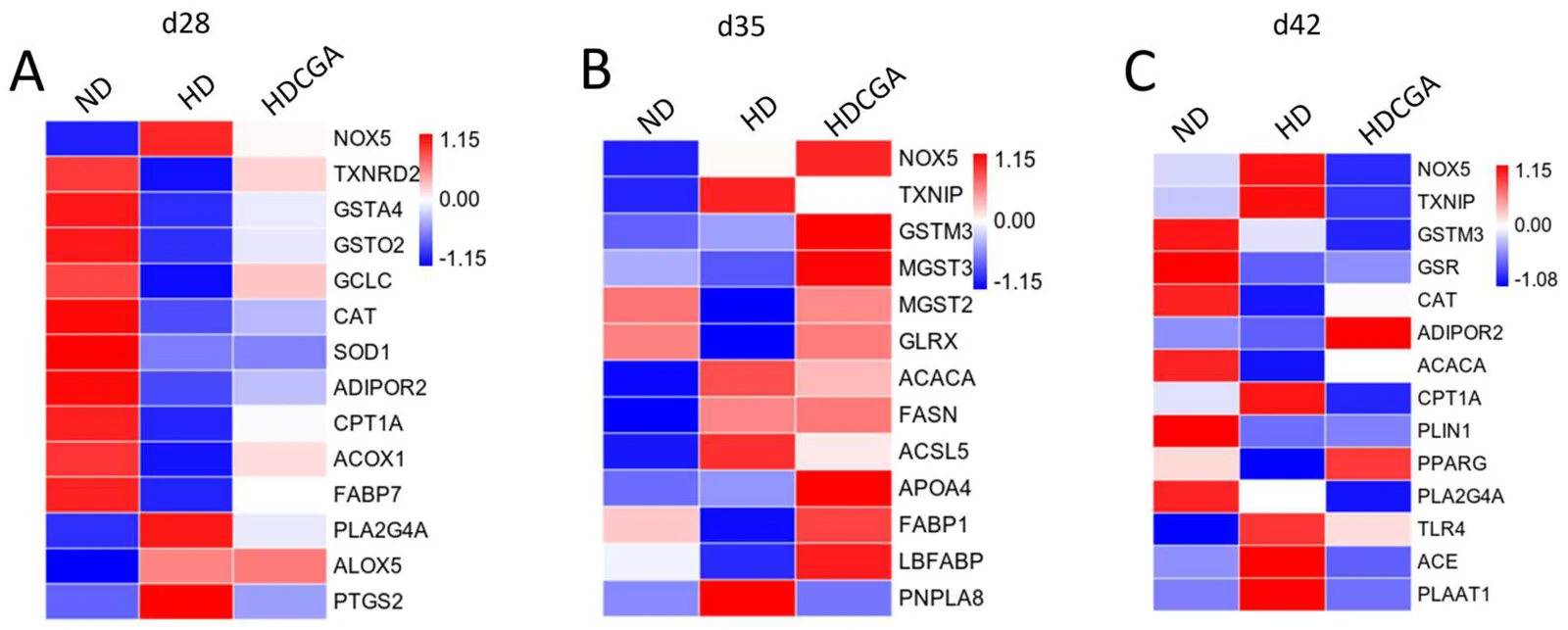
Expression patterns of selected hepatic DEGs related to redox regulation, lipid metabolism, and inflammation (**A**–**C**). Expression values were Z-score normalized. Red indicates relatively higher transcript abundance, and blue indicates relatively lower transcript abundance. ND, normal stocking density with the basal diet; HD, high stocking density with the basal diet; HDCGA, high stocking density with 0.1% chlorogenic acid; d28, d35, and d42, 28, 35, and 42 d of age, respectively. RNA-seq analyses used *n* = 4 biological replicates per represented group per age.

Representative DEGs associated with redox regulation, lipid metabolism, and inflammation were visualized by heatmap (Figure 4). At 28 d, the HD group showed a higher expression of NOX5, PLA2G4A, PTGS2 (COX-2), and ALOX5, and a lower expression of several antioxidant and fatty-acid-oxidation genes than the ND group. At 35 d, the HD group was associated with higher NOX5, TXNIP, ACACA, FASN, ACSL5, and PNPLA8 expression and lower expression of glutathione-related genes and FABP1. At 42 d, NOX5, TXNIP, TLR4, ACE, and PLAAT1 remained relatively high, whereas GSTM3, GSR, CAT, PLIN1, and PPARG were relatively low. In the HDCGA group, several of these expression patterns shifted toward those observed in the ND group relative to the HD group. Because the heatmap displays normalized transcript abundance, these results describe relative expression patterns and do not establish corresponding changes in protein abundance or enzyme activity.

### 3.5. KEGG Pathway Enrichment Profiles of DEGs

**Figure 5:**
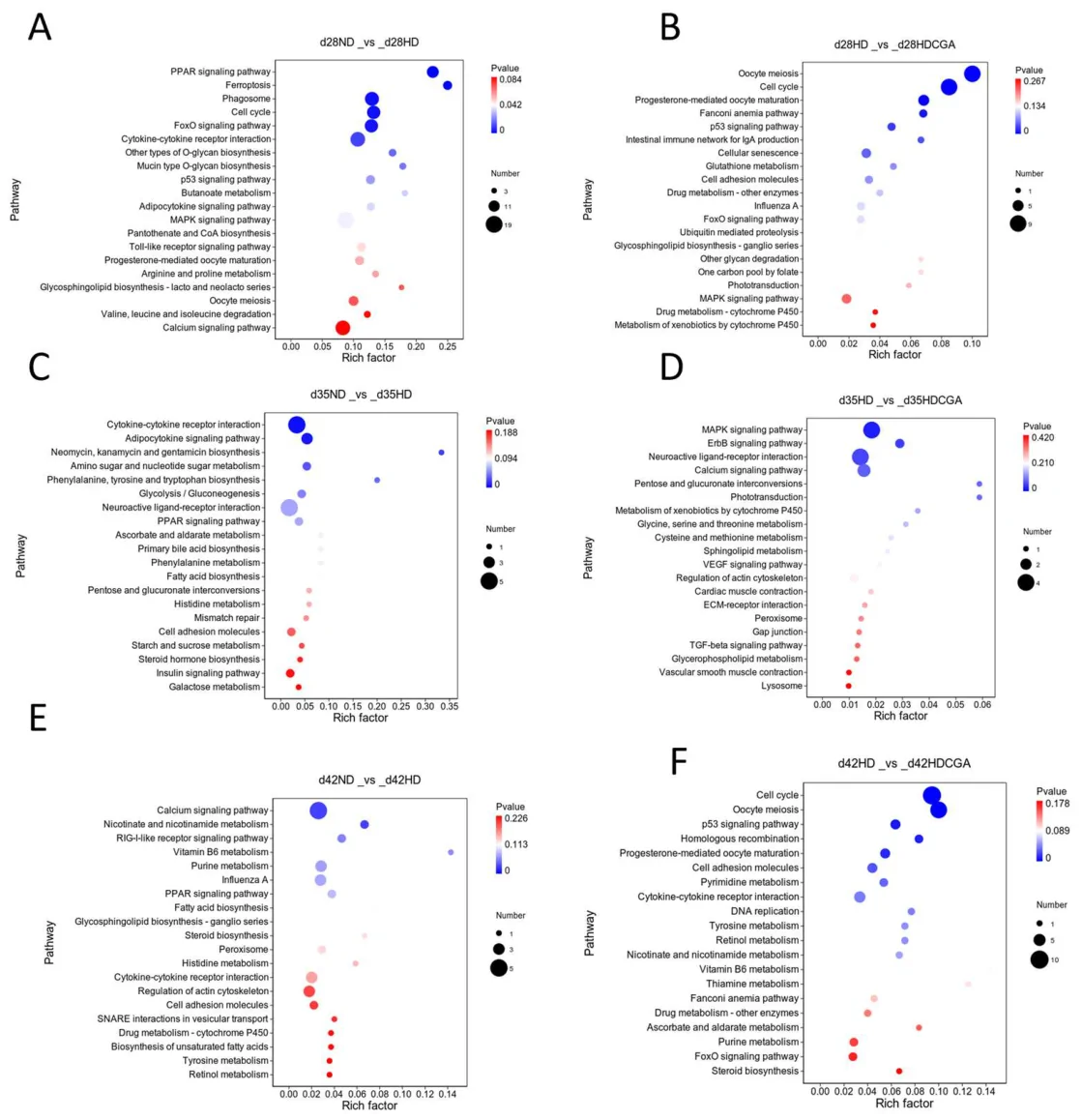
KEGG pathway enrichment profiles of hepatic DEGs in broilers under HSD. Panels (**A**–**F**) show ND versus HD and HD versus HDCGA comparisons at 28, 35, and 42 d. Bubble size represents the number of DEGs assigned to a pathway, and bubble color represents the enrichment *p* value reported by DAVID. Displayed pathways that did not meet an adjusted *p* < 0.05 are interpreted descriptively. ND, normal stocking density with the basal diet; HD, high stocking density with the basal diet; HDCGA, high stocking density with 0.1% chlorogenic acid. RNA-seq analyses used *n* = 4 biological replicates per represented group per age.

KEGG pathway enrichment profiles are shown in Figure 5. The plots display the highest-ranked DAVID outputs, but not every displayed pathway met the adjusted *p* threshold of 0.05; pathways that did not meet this threshold are presented descriptively. In the ND versus HD comparison, the top-ranked pathways at 28 d included PPAR, FoxO, and adipocytokine signaling. At 35 d, they included PPAR signaling, adipocytokine signaling, and fatty-acid biosynthesis. At 42 d, the top-ranked pathways included calcium signaling, nicotinate and nicotinamide metabolism, PPAR signaling, fatty-acid biosynthesis, peroxisome, and unsaturated-fatty-acid biosynthesis. In the HD versus HDCGA comparison, the top-ranked pathways at 28 d included glutathione metabolism, FoxO signaling, one-carbon metabolism, cell-adhesion molecules, and MAPK signaling. At 35 d, they included calcium signaling, amino acid metabolism, peroxisome, and glycerophospholipid metabolism. At 42 d, they included nicotinate and nicotinamide metabolism, vitamin B_6_ and thiamine metabolism, FoxO signaling, steroid biosynthesis, and cell-adhesion molecules. These profiles identify treatment-associated pathway patterns but do not, by themselves, demonstrate pathway activation or inhibition.

### 3.6. RT-qPCR Validation of Gene Expression

**Figure 6:**
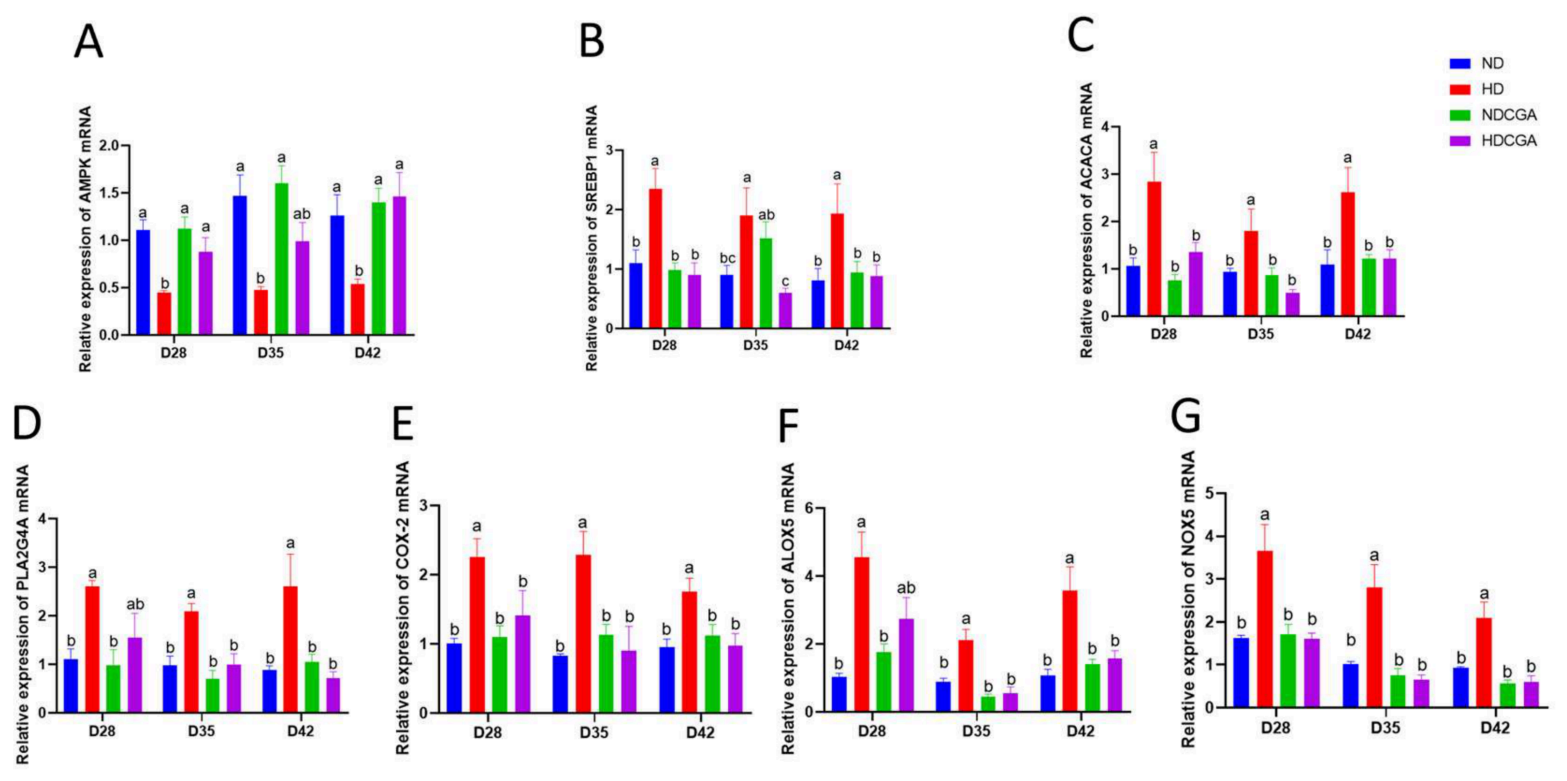
Relative hepatic mRNA abundance of (**A**) PRKAA1 (AMPK), (**B**) SREBF1 (SREBP1), (**C**) ACACA, (**D**) PLA2G4A, (**E**) PTGS2 (COX-2), (**F**) ALOX5, and (**G**) NOX5 at 28, 35, and 42 d. Values are means ± SEM (*n* = 6 biological replicates per treatment per age; one sample per replicate cage). Different letters indicate significant differences among treatments at the same age (*p* < 0.05). ND, normal stocking density with the basal diet; HD, high stocking density with the basal diet; NDCGA, normal stocking density with 0.1% chlorogenic acid; HDCGA, high stocking density with 0.1% chlorogenic acid.

RT-qPCR was used to evaluate selected genes associated with redox regulation, lipid synthesis, and arachidonic acid metabolism (Figure 6). Compared with the ND group, the HD group showed lower hepatic AMPK mRNA abundance and higher SREBP1, ACACA, PLA2G4A, COX-2, ALOX5, and NOX5 mRNA abundance at 28, 35, and 42 d (*p* < 0.05). Compared with the HD group, the HDCGA group showed higher AMPK mRNA abundance at 28 and 42 d and lower SREBP1, ACACA, PLA2G4A, COX-2, ALOX5, and NOX5 mRNA abundance across the evaluated ages (*p* < 0.05). These transcript-level findings were directionally consistent with the biochemical and histological observations but should not be interpreted as direct measurements of protein abundance or enzyme activity.

## 4. Discussion

Increasing the bird-number density from 14 to 22 birds/m^2^ increased the recorded live-weight load from approximately 35 to 50 kg/m^2^ by 42 d. Based on the verified experimental-cage records, the high-density groups had lower body weights from 21 d onward and lower ADG during days 35–42 and 21–42 than their corresponding normal-density groups. The absence of a significant ADG difference during days 21–28 and 28–35 suggests that the growth penalty became more evident as live-weight density increased in the later growth period. CGA supplementation from 7 d of age did not significantly restore body weight or ADG within either stocking density. Thus, the hepatic differences observed after CGA supplementation should not be interpreted as a demonstrated growth-promoting effect in this experiment. Rather, the HDCGA group showed partial attenuation of several hepatic biochemical, histological, and transcript-level abnormalities relative to the HD group despite limited measurable recovery of growth.

HSD has previously been associated with reduced antioxidant defense and increased lipid peroxidation in broilers [1,10,11]. Oxidative stress is also a central contributor to hepatic cellular injury and tissue remodeling, and avian stress models demonstrate coordinated disruption of pro-oxidant and antioxidant systems [30,31,32,33,34]. In the present study, the biochemical antioxidant indices did not differ significantly among treatments at 21 or 28 d, although qualitative histological and transcript-level alterations were already evident at 28 d. More consistent biochemical impairment emerged at 35 and 42 d, when the HD group had lower T-AOC, SOD, and CAT activities and higher MDA content than the ND group. The persistently higher NOX5 mRNA abundance in HD broilers was temporally associated with this later redox imbalance. Compared with the HD group, the HDCGA group showed higher antioxidant-enzyme activities, lower MDA, and lower NOX5 mRNA abundance at relevant ages. CGA has radical-scavenging properties attributable in part to its catechol structure [17], and poultry studies also associate CGA with enhancements in endogenous antioxidant systems [21,22,35]. Nevertheless, ROS production and NOX5 enzyme activity were not directly measured; therefore, the present data do not establish NOX5-derived ROS generation or its inhibition by CGA.

The liver is the principal site of de novo fatty-acid synthesis in poultry [36], making it particularly susceptible to stress-induced changes in lipid allocation. Representative HD sections showed progressively greater visible lipid droplet deposition, and the transcriptomic analysis identified treatment-associated enrichment patterns involving fatty-acid biosynthesis and PPAR signaling. AMPK/PPAR-associated regulation has been demonstrated in chicken hepatocytes and broilers [37,38]. More broadly, AMPK is a key energy sensor that can limit lipid synthesis and support fatty-acid oxidation [39,40]. In the present study, HD was associated with lower AMPK mRNA abundance and higher SREBP1 and ACACA mRNA abundance, whereas the HDCGA group showed shifts in the opposite direction together with less visible lipid droplet deposition in representative sections. Previous experimental evidence indicates that CGA can influence AMPK-associated metabolic regulation and suppress lipogenic transcription [23,41,42]. The current data are therefore consistent with modulation of an AMPK–SREBP1–ACACA-related transcriptional program. However, AMPK phosphorylation, SREBP1 processing, ACACA activity, quantitative lipid histomorphometry, and lipid flux were not measured, and the proposed sequence should be regarded as a working model rather than a demonstrated signaling cascade.

Oxidative membrane damage and lipid overload can also amplify inflammatory lipid signaling. PLA2G4A releases arachidonic acid from membrane phospholipids, after which COX-2 and ALOX5 generate downstream eicosanoid mediators [43,44,45]. Related avian studies also link fatty-acid signaling with arachidonate-lipoxygenase expression and hepatic lipid metabolism [46,47]. The HD group showed higher PLA2G4A, COX-2, and ALOX5 mRNA abundance together with inflammatory cell infiltration in representative sections, whereas the HDCGA group exhibited a lower abundance of these transcripts and a less pronounced inflammatory histological appearance. These observations are compatible with a relationship among oxidative/lipid stress and arachidonic-acid-related inflammatory transcription, but they do not establish causal ordering or direct pathway inhibition by CGA. Because arachidonic acid release, PGE_2_, LTB_4_, and the relevant enzyme activities were not quantified, the findings indicate attenuation of a PLA2G4A/COX-2/ALOX5-related transcriptional signature rather than direct measurement of eicosanoid synthesis.

AST, ALT, and ALP activities were measured in liver homogenates rather than serum and therefore describe treatment-associated changes in hepatic tissue enzyme activity, not circulating diagnostic markers of hepatocellular leakage or biliary dysfunction. At 35 d, the HD group had higher AST, ALT, and ALP activities than the ND group. At 42 d, the HD group had higher AST and ALP activities than the ND group, whereas the overall treatment effect for ALT was borderline (*p* = 0.053) and was not interpreted as statistically significant. Compared with the HD group, the HDCGA group had lower AST and ALP activities at 35 and 42 d and lower ALT activity at 35 d. Considered together with the qualitative histological observations, these changes are consistent with an altered hepatic status, but they should not be interpreted as stand-alone evidence of clinical liver dysfunction [48].

**Figure 7:**
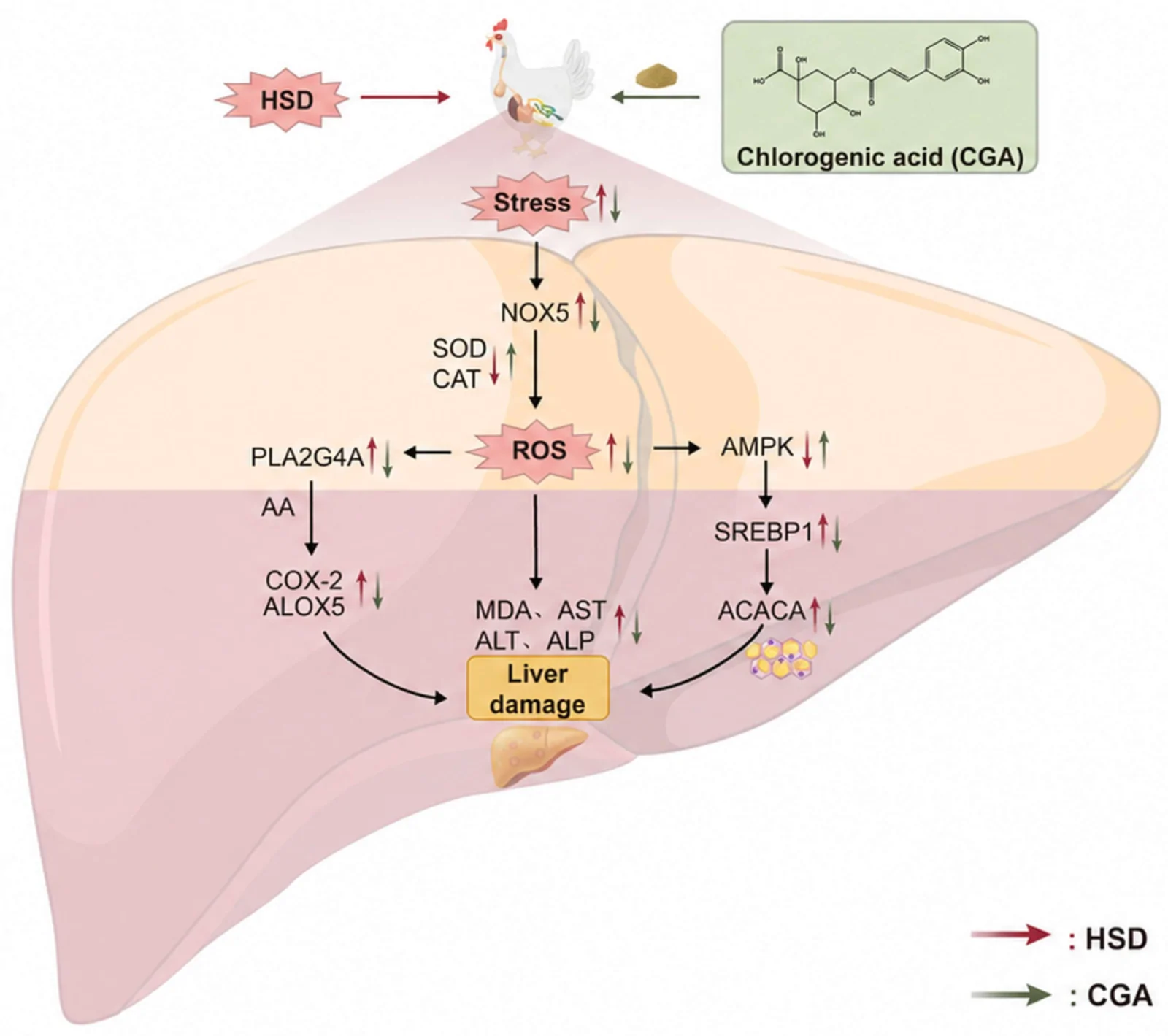
Working model of treatment-associated hepatic changes in broilers under HSD and dietary CGA supplementation. The HD treatment was associated with higher NOX5 mRNA abundance and oxidative pressure, lower AMPK mRNA abundance, higher SREBP1/ACACA-related lipogenic transcription, and greater PLA2G4A/COX-2/ALOX5-related inflammatory transcription. Relative to HD, the HDCGA group was associated with shifts in these transcript and biochemical patterns toward improved redox and metabolic homeostasis. Red arrows indicate HD-associated changes, and green arrows indicate HDCGA-associated changes relative to HD. The diagram is a hypothesis-generating synthesis based on transcript abundance, biochemical indices, and qualitative histological observations; it does not demonstrate NOX5-derived ROS production, protein-level pathway activation, causal pathway ordering, or quantitative histomorphometric effects. ND, normal stocking density with the basal diet; HD, high stocking density with the basal diet; NDCGA, normal stocking density with 0.1% chlorogenic acid; HDCGA, high stocking density with 0.1% chlorogenic acid.

Taken together, the results support an integrated working model in which the high-density treatment is associated with progressively greater hepatic oxidative and metabolic pressure, whereas the HDCGA group shows partial normalization of several biochemical and transcript-level readouts relative to the HD group. Potential contributors include the known radical-scavenging properties of CGA and treatment-associated differences in antioxidant, AMPK-related, lipogenic, and arachidonic-acid-related transcripts. However, causal ordering among these processes cannot be established from the present data. The study measured mRNA abundance rather than protein abundance, phosphorylation, pathway-specific enzyme activity, or metabolic flux, and the histological observations were qualitative rather than quantitative. Figure 7 is therefore hypothesis-generating and requires confirmation through protein-level, functional, and quantitatively scored histological experiments.

## 5. Conclusions

Compared with their corresponding normal-density groups, the high-density groups had lower body weights and later-stage average daily gains and showed progressive disturbances in hepatic redox balance, lipid homeostasis, inflammatory status, and selected hepatic tissue enzyme activities (Figure 7). Relative to the HD group, dietary supplementation with 0.1% CGA from 7 d of age was associated with higher antioxidant capacity indices, less visible lipid droplet deposition and inflammatory cell infiltration in representative sections, and partial normalization of selected hepatic tissue enzyme activities, but it did not significantly restore body weight or average daily gain. The transcript-level findings were consistent with lower NOX5 mRNA abundance, an altered AMPK–SREBP1–ACACA-related lipogenic transcriptional pattern, and attenuation of a PLA2G4A/COX-2/ALOX5-related inflammatory transcriptional signature in the HDCGA group relative to HD. These mechanistic interpretations remain provisional because protein abundance, phosphorylation, pathway-specific enzyme activity, metabolic flux, and quantitative histomorphometry were not directly measured. Because only one CGA inclusion level was evaluated, the present study does not establish a dose–response relationship, a comprehensive safety profile, or routine commercial efficacy. CGA may nevertheless warrant further evaluation as a liver-health-supporting nutritional strategy under high-density conditions.

## Figures and Tables

**Table 1:** Composition and nutrient levels of the basal diets (%).

Item	1–21 d	22–42 d
***Ingredients***		
Corn (CP 7.9%)	58.21	62.09
Soybean meal (CP 44.3%)	33.11	31.62
Fish meal (CP 62.5%)	2.00	0.00
Soybean oil	2.50	2.50
CaCO_3_	1.30	1.20
CaHPO_4_	1.65	1.70
NaCl	0.40	0.30
Lys-HCl	0.08	0.02
DL-Met	0.26	0.17
L-Thr	0.02	0.01
Choline chloride	0.15	0.10
Premix ^1^	0.20	0.00
Premix ^2^	0.00	0.20
Displayed ingredient sum	99.88	99.91
Nominal formulation total	100.00	100.00
***Nutrient composition*** **^3^**		
Metabolizable energy, MJ/kg	12.47	12.53
Crude protein	20.92	19.27
Total Ca	1.01	0.91
Total *p*	0.72	0.67
Available *p*	0.45	0.40
Lys	1.19	1.00
Met	0.58	0.46
Met + Cys	0.91	0.77
Thr	0.81	0.72
Val	0.95	0.87
Trp	0.23	0.21

^1^ Premix 1 was included during days 1–21 and provided per kilogram of diet: Cu, 8 mg; Fe, 100 mg; Mn, 120 mg; Zn, 100 mg; I, 0.7 mg; vitamin A, 8000 IU; vitamin D_3_, 2000 IU; vitamin E, 20 IU; vitamin K_3_, 3.2 mg; vitamin B_1_, 2 mg; vitamin B_2_, 6.4 mg; vitamin B_6_, 4 mg; vitamin B_12_, 0.2 mg; D-biotin, 1.1 mg; D-pantothenic acid, 12 mg; folic acid, 1 mg; and nicotinamide, 40 mg. ^2^ Premix 2 was included during days 22–42 and provided per kilogram of diet: Cu, 8 mg; Fe, 80 mg; Mn, 100 mg; Zn, 80 mg; I, 0.7 mg; vitamin A, 6000 IU; vitamin D_3_, 1500 IU; vitamin E, 15 IU; vitamin K_3_, 2.4 mg; vitamin B_1_, 1.5 mg; vitamin B_2_, 4.8 mg; vitamin B_6_, 3 mg; vitamin B_12_, 0.15 mg; D-biotin, 0.825 mg; D-pantothenic acid, 9 mg; folic acid, 0.75 mg; and nicotinamide, 30 mg. ^3^ Crude protein, total calcium, and total phosphorus were analytically measured. Metabolizable energy and amino acid concentrations were calculated; amino acid values represent total rather than standardized ileal digestible concentrations. Ingredient inclusion values are reproduced from the archived formulation to two decimal places. The displayed ingredient sums are 99.88% and 99.91%, whereas the nominal formulation total was 100.00% in both phases; the small residual difference was not redistributed among individual ingredients. Table 1 describes the basal diets. Treatment-specific CGA supplementation (0.1% from 7 to 42 d in NDCGA and HDCGA groups) is described in Section 2.1 and is not included in the basal-diet ingredient totals.

**Table 2:** Primer sequences used for real-time PCR.

Gene	Primer	Primer Sequence (5′–3′)	GenBank Number
*PRKAA1* (*AMPK*)	Forward	CTGTCACAGGCACATGGTAGT	NM_001039603.2
	Reverse	TGGGCCTGCATATAACCTTCC	
*ACACA*	Forward	AATGGCAGCTTTGGAGGTGT	NM_205505.2
	Reverse	TTCTGTTTGGGTGGGAGGTG	
*SREBF1* (*SREBP1*)	Forward	GAGACCATCTACAGCTCCGC	NM_204126.3
	Reverse	CATCCGAAAAGCACCCCTCT	
*PLA2G4A*	Forward	TGTTTGCAGACTGGGTGGAA	NM_205423.1
	Reverse	TGCCCCAGACACCCATTAAG	
*ALOX5*	Forward	GCCTTAACGGGTTTCAGGTTG	XM_040675252.2
	Reverse	TCCAGCGGTAACATGGGAAT	
*PTGS2* (*COX-2*)	Forward	CCTGGAGAAAGGAGACCTCG	NM_001167718.1
	Reverse	TGGGTAACAGCAGTCACCAG	
*NOX5*	Forward	AGCCATCGAACTGACGTTGT	NM_001305472.4
	Reverse	GCCCATCGATGTAGCACTTG	
*GAPDH*	Forward	TCGGAGTCAACGGATTTGGC	NM_205344.2
	Reverse	TTCCCGTTCTCAGCCTTGAC	

**Table 3:** Effects of CGA on body weight and average daily gain of broilers under HSD.

Item	ND	HD	NDCGA	HDCGA	SEM	Treatment *p* Value
Body weight, 21 d	558.73 ^a^	511.35 ^bc^	544.83 ^ab^	494.78 ^c^	12.166	0.0048
Body weight, 28 d	1083.35 ^a^	1015.22 ^bc^	1054.67 ^ab^	982.37 ^c^	13.847	0.0003
Body weight, 35 d	1760.46 ^a^	1620.53 ^b^	1744.79 ^a^	1606.92 ^b^	29.449	0.0015
Body weight, 42 d	2500.33 ^a^	2284.07 ^b^	2463.17 ^a^	2244.76 ^b^	42.553	0.0006
ADG, 21–28 d	74.95	71.98	72.83	69.65	2.075	0.3675
ADG, 28–35 d	96.73	86.47	98.59	89.22	4.696	0.2354
ADG, 35–42 d	105.70 ^a^	94.79 ^bc^	102.62 ^ab^	91.12 ^c^	2.810	0.0051
ADG, 21–42 d	92.46 ^a^	84.42 ^b^	91.35 ^a^	83.33 ^b^	1.812	0.0027

Body weight is expressed as g/bird and ADG as g/bird/day. ADG, average daily gain; SEM, standard error of the mean. ND, normal stocking density with basal diet; HD, high stocking density with basal diet; NDCGA, normal stocking density with 0.1% CGA from day 7; HDCGA, high stocking density with 0.1% CGA from day 7. *n* = 6 cages; different superscripts letters indicate *p* < 0.05.

**Table 4:** Effects of CGA on hepatic antioxidant indices in broilers under HSD.

Item	Age	ND	HD	NDCGA	HDCGA	SEM	Treatment *p* Value
T-AOC (μmol/g)	21 d	99.92	95.27	101.87	99.78	1.769	0.629
T-AOC (μmol/g)	28 d	107.17	88.89	110.41	102.08	2.700	0.062
T-AOC (μmol/g)	35 d	111.63 ^a^	94.88 ^b^	112.21 ^a^	104.33 ^a^	2.020	0.002
T-AOC (μmol/g)	42 d	104.08 ^a^	81.71 ^b^	103.68 ^a^	99.23 ^a^	2.178	<0.0001
SOD (U/mg protein)	21 d	58.74	56.70	58.98	59.45	0.902	0.741
SOD (U/mg protein)	28 d	55.06	52.80	55.60	54.74	0.806	0.523
SOD (U/mg protein)	35 d	59.12 ^a^	53.45 ^b^	60.89 ^a^	56.71 ^ab^	0.902	0.012
SOD (U/mg protein)	42 d	64.42 ^a^	52.76 ^b^	61.19 ^a^	64.24 ^a^	1.624	0.024
CAT (U/mg protein)	21 d	44.36	43.52	46.12	45.84	0.702	0.540
CAT (U/mg protein)	28 d	41.07	34.73	41.97	39.21	0.951	0.083
CAT (U/mg protein)	35 d	39.12 ^a^	34.06 ^b^	39.70 ^a^	38.08 ^a^	0.674	0.005
CAT (U/mg protein)	42 d	34.47 ^a^	30.73 ^b^	34.90 ^a^	33.92 ^a^	0.378	<0.0001
MDA (nmol/g protein)	21 d	509.31	545.50	521.81	535.20	8.593	0.497
MDA (nmol/g protein)	28 d	446.91	477.12	445.96	451.84	6.997	0.366
MDA (nmol/g protein)	35 d	470.81 ^b^	544.40 ^a^	455.25 ^b^	462.57 ^b^	9.046	<0.0001
MDA (nmol/g protein)	42 d	493.03 ^c^	588.23 ^a^	474.15 ^c^	533.57 ^b^	10.796	<0.0001

T-AOC, total antioxidant capacity; SOD, superoxide dismutase; CAT, catalase; MDA, malondialdehyde; SEM, standard error of the mean; ND, normal stocking density with the basal diet; HD, high stocking density with the basal diet; NDCGA, normal stocking density with 0.1% chlorogenic acid; HDCGA, high stocking density with 0.1% chlorogenic acid. Different superscript letters within an age indicate *p* < 0.05.

**Table 5:** Effects of CGA on hepatic tissue enzyme activities in broilers under HSD.

Item	Age	ND	HD	NDCGA	HDCGA	SEM	Treatment *p* Value
AST (U/g protein)	21 d	177.74	152.93	177.72	167.99	4.755	0.330
AST (U/g protein)	28 d	163.41	169.73	143.34	166.46	5.151	0.401
AST (U/g protein)	35 d	104.16 ^c^	148.80 ^a^	92.77 ^d^	117.86 ^b^	6.161	<0.001
AST (U/g protein)	42 d	130.03 ^b^	146.87 ^a^	128.50 ^b^	124.08 ^b^	2.736	<0.001
ALT (U/g protein)	21 d	50.80	55.29	55.32	54.46	1.592	0.816
ALT (U/g protein)	28 d	63.35	67.92	67.08	70.64	1.528	0.474
ALT (U/g protein)	35 d	61.58 ^b^	76.91 ^a^	68.38 ^b^	66.61 ^b^	1.922	0.012
ALT (U/g protein)	42 d	56.09	76.13	58.32	63.32	2.772	0.053
ALP (U/g protein)	21 d	55.26	54.03	53.75	64.34	1.893	0.246
ALP (U/g protein)	28 d	61.18	51.35	55.24	55.40	1.867	0.402
ALP (U/g protein)	35 d	50.45 ^b^	75.78 ^a^	58.64 ^b^	57.02 ^b^	3.317	0.011
ALP (U/g protein)	42 d	60.12 ^b^	68.86 ^a^	57.71 ^b^	57.83 ^b^	1.591	0.037

AST, aspartate aminotransferase; ALT, alanine aminotransferase; ALP, alkaline phosphatase; SEM, standard error of the mean; ND, normal stocking density with the basal diet; HD, high stocking density with the basal diet; NDCGA, normal stocking density with 0.1% chlorogenic acid; HDCGA, high stocking density with 0.1% chlorogenic acid. Different superscript letters within an age indicate *p* < 0.05.

## Data Availability

The data supporting the findings of this study are available from the corresponding author upon reasonable request. The raw RNA-sequencing data are being prepared for deposition in an appropriate public repository in accordance with the journal’s data-sharing requirements; the verified accession number will be added to the manuscript immediately after assignment.
